# The Effect of Non-invasive Spinal Cord Stimulation on Anorectal Function in Individuals With Spinal Cord Injury: A Case Series

**DOI:** 10.3389/fnins.2022.816106

**Published:** 2022-02-17

**Authors:** Evgeniy Kreydin, Hui Zhong, Igor Lavrov, V. Reggie Edgerton, Parag Gad

**Affiliations:** ^1^Keck School of Medicine, Institute of Urology, University of Southern California, Los Angeles, CA, United States; ^2^Rancho Los Amigos National Rehabilitation Center, Rancho Research Institute, Downey, CA, United States; ^3^SpineX Inc., Los Angeles, CA, United States; ^4^Department of Neurobiology, University of California, Los Angeles, Los Angeles, CA, United States; ^5^Department of Neurology, Department of Biomedical Engineering, Mayo Clinic, Rochester, NY, United States; ^6^Institute of Fundamental Medicine and Biology, Kazan Federal University, Kazan, Russia; ^7^Department of Neurosurgery, University of California, Los Angeles, Los Angeles, CA, United States; ^8^Brain Research Institute, University of California, Los Angeles, Los Angeles, CA, United States; ^9^Institut Guttmann, Hospital de Neurorehabilitació, Institut Universitari Adscrit a la Universitat Autònoma de Barcelona, Barcelona, Spain

**Keywords:** non-invasive spinal cord stimulation, spinal cord injury, stroke, multiple sclerosis, neurogenic bladder, overactive bladder urodynamics

## Abstract

Spinal cord injury (SCI) is a devastating condition that impacts multiple organ systems. Neurogenic bowel dysfunction (NBD) frequently occurs after a SCI leading to reduced sensation of bowel fullness and bowel movement often leading to constipation or fecal incontinence. Spinal Neuromodulation has been proven to be a successful modality to improve sensorimotor and autonomic function in patients with spinal cord injuries. The pilot data presented here represents the first demonstration of using spinal neuromodulation to activate the anorectal regions of patients with spinal cord injuries and the acute and chronic effects of stimulation. We observed that spinal stimulation induces contractions as well as changes in sensation and pressure profiles along the length of the anorectal region. In addition, we present a case report of a patient with a SCI and the beneficial effect of spinal neuromodulation on the patient’s bowel program.

## Introduction

Neurogenic bowel dysfunction (NBD) is a significant source of morbidity after a spinal cord injury (SCI). After an SCI, many patients develop the inability to empty the bowels spontaneously and become reliant on onerous and time-consuming bowel programs to manage constipation and stool retention. Others may develop stool incontinence secondary to obstipation or anal sphincter weakness. As a result, NBD has a marked deleterious effect on patients’ health and quality of life. Indeed, several survey studies of SCI patients have demonstrated that recovery of bowel function is one of the top rehabilitation priorities in this population (Anderson, 2004; Bloemen-Vrencken et al., 2005; Ditunno et al., 2008).

Despite such significant impact on a patient’s life, treatment options for NBD are limited. Rectal suppositories and oral stool softeners and laxatives are the mainstays of therapy but have a limited efficacy in decreasing the time devoted to bowel programs and do little to normalize bowel function and stool transit. Surgical interventions, such as creation of colostomy or Malone Antegrade Continence Enema (MACE) are morbid and can have a negative impact on an already-impaired body image in patients with SCI (Waddell et al., 2020). More recently, neuromodulation has begun to be explored as a therapy for NBD. Techniques such as functional electrical stimulation, magnetic stimulation, sacral nerve stimulation, dorsal genital nerve stimulation and transcutaneous interferential electrical stimulation have been attempted as a therapy for NBD in adults (SCI) and children (myelomeningocele) with promising results (Deng et al., 2018; Parittotokkaporn et al., 2020).

We have developed and implemented a novel Spinal Cord Neuromodulator (SCONE™, SpineX Inc., Los Angeles, CA, United States) as a non-invasive stimulation modality to facilitate functional recovery after SCI. This approach delivers an electrical stimulation to the spinal cord without eliciting significant cutaneous discomfort by incorporating a high-frequency carrier current. We have previously demonstrated that non-invasive spinal cord neuromodulation can improve lower (Gad et al., 2015; Gerasimenko Y. P. et al., 2015; Parag Gad and Reggie Edgerton, 2019), upper extremity (Gad P. et al., 2018; Inanici et al., 2018), trunk stability (Rath et al., 2018), respiratory (Gad et al., 2020) and lower urinary tract function (Gad P. N. et al., 2018; Kreydin et al., 2020; Gad et al., 2021b) in patients with SCI. In this study, we used anorectal manometry (ARM) to determine the effect of acute SCONE™ stimulation on anorectal physiology in three chronically paralyzed patients and the effect of chronic stimulation on bowel program times in one patient.

## Materials and Methods

### Patient Recruitment

This study was approved by the Institutional Review Board of Rancho Research Institute, the research arm of Rancho Los Amigos National Rehabilitation Center, Downey, CA, United States. The research participants signed an informed consent form before the start of the study and consented to the data being used in future publications and presentations. The patient demographics and injury characteristics were as follow: Acute Stimulation Patient 1 (ASP1) was a 39-year-old male who sustained a SCI at C5 (AIS A) 9 years prior to study. ASP2 was a 29-year-old male who sustained a SCI at T6 (AIS A) 5 years prior to the study. ASP3 was a 28-year-old female who sustained a SCI at T6 (AIS A) 2 years prior to the study. Finally, the patient who underwent chronic stimulation [Chronic Stimulation Patient 1 (CSP1)] was a 32-year-old female who sustained a SCI at T6 (AIS A) 2 years prior to the study.

### Spinal Stimulation

**FIGURE 1:**
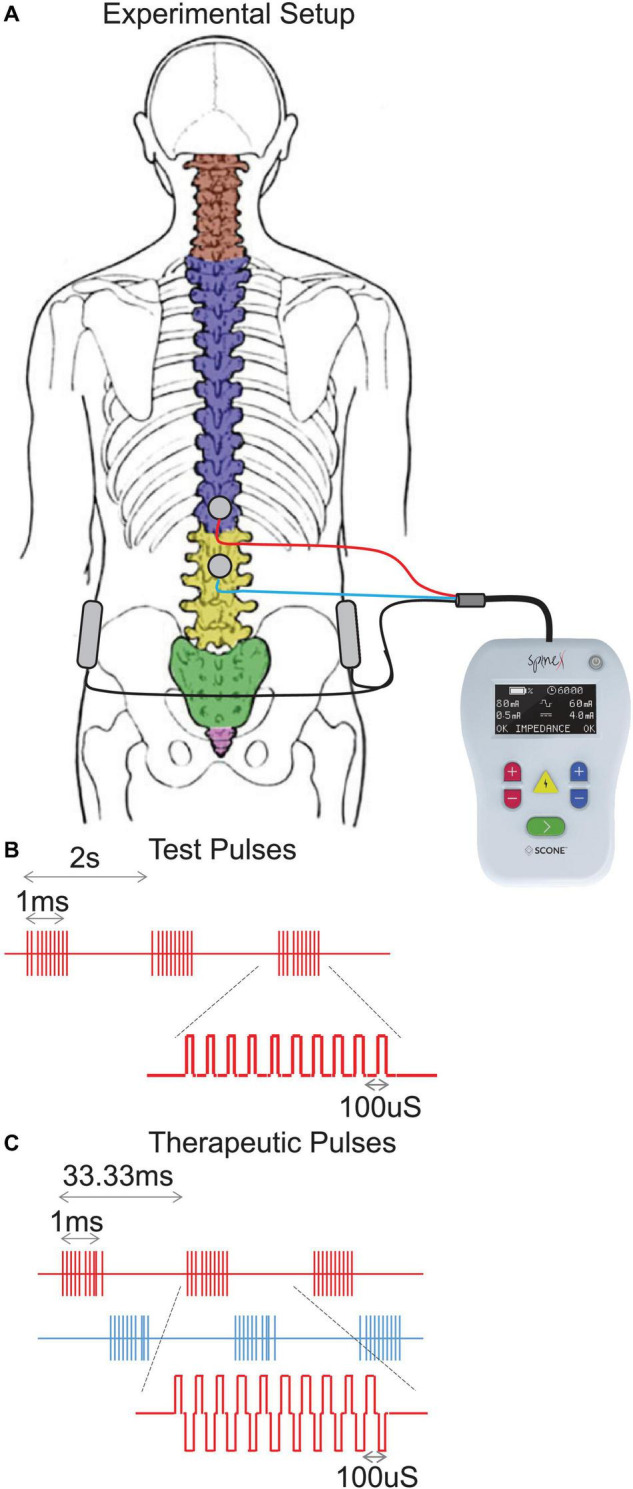
**(A)** Experimental setup demonstrating the stimulation electrodes over T11-12 and L1-2 vertebral levels and return electrodes over the iliac crests. **(B)** Test pulses showing the train of monophasic pulses including the high frequency (10 KHz) pulses on for a period of 1 ms and low frequency (0.5 Hz, repeating every 2 s) pulses and **(C)** Therapeutic pulses showing the train of biphasic pulses including the high frequency (10 KHz) pulse on for a period of 1 ms and low frequency (30 Hz, repeating every 33.33 ms) pulses.

Spinal stimulation was delivered using a proprietary SCONE™ device (SpineX Inc., Los Angeles, CA, United States). The stimulation was delivered either as single test pulses or as therapeutic pulses. The test pulse waveform consisted of a monophasic pulse at 0.5 Hz with a high frequency carrier frequency (10 KHz) with a pulse width of 1 ms. The therapeutic waveform consisted of two alternating pulses of opposite polarities separated by a 1 uS delay forming a delayed biphasic waveform. The pulses consisted of a high frequency biphasic carrier pulse (10 KHz) combined with a low frequency (30 Hz) burst pulse each with a pulse width of 1 ms. Stimulation was applied using an adhesive electrode over the interspinous ligaments of T11 and L1 serving as the cathode and two adhesive electrodes over the iliac crests as the anodes (Figure 1). The intensity of stimulation during acute mapping studies (0.5 Hz) were set at a supra sensory and motor threshold, i.e., the lowest intensity at which contractions were visible on the pressure probes. At this intensity all patients also noted contractions of several lower extremity muscles. The intensity of stimulation during therapeutic stimulation (30 Hz) was set at a supra-sensory and sub-motor threshold level, i.e., at 80% of the intensity that generated a visible contraction on the pressure probes. At this intensity, patients could feel the stimulation but did not note any lower extremity muscles contractions and tolerated the stimulation well.

### Anorectal Manometry and Mapping

Acute stimulation patients were asked to perform an enema at home at least 6 h prior to the study. Anorectal manometry (ARM) probe was placed in the anorectal region. The probe consisted of four sensors around the periphery of the tip. A balloon was positioned at the proximal end of the catheter. The anorectum was mapped by positioning the catheter at specific distances from the anal verge starting at 2 cm and continuing up to 10 cm in increments of 1 cm. At each catheter location, stimulation test pulses were delivered starting at 10 mA and increasing to 200 mA in increments of 10 mA. A minimum of five pulses were delivered at each intensity. A minimum of 3 mins pause was taken prior to moving the catheter to the next position. Anorectal profiles were created by slowly withdrawing the catheter at a constant speed of 0.5–1 cm/s. These profiles were created with the patient in a relaxed position (relax) and while attempting to generate a bowel movement (squeeze) on command. Following test stimulation, tonic submotor stimulation was applied and anorectal pressure profiles were generated in an identical manner. Finally, to test anorectal sensation, the catheter was placed at 5 cm from the anal verge, the balloon was slowly filled with air using an external syringe at a rate of 60 ml/min without and with sub-motor threshold stimulation. The patient was asked to report when they experienced rectal sensation and the 1st volume, at which this occurred, was recorded.

### Chronic Spinal Stimulation

The chronic stimulation patient (CSP1) was recruited to study the effect of chronic spinal stimulation on bowel function. This patient needed external digital stimulation to assist bowel movement and relied on rectal suppositories to move her bowels every 1–2 days. She was asked to monitor the duration of her bowel program for the duration of the study. The therapeutic waveform consisted of two alternating pulses of opposite polarities separated by a 1 uS delay forming a delayed biphasic waveform. The pulses consisted of a high frequency biphasic carrier pulse (10 KHz) combined with a low frequency (30 Hz) burst pulse each with a pulse width of 1 ms (Figure 1). Stimulation was applied using an adhesive electrode over the interspinous ligaments of T11 and L1 serving as the cathode and two adhesive electrodes over the iliac crests as the anodes. She received 1 h of stimulation 5 days a week for 5 weeks. On the days that the patient had a bowel program, she completed the bowel program 1–3 h prior to receiving stimulation. For the first 18 days, stimulation was delivered at a therapeutic level whereas during the last 18 days, stimulation intensity was reduced to an amplitude not expected to generate any physiological response based on our previous studies (sham stimulation). The patient was blinded by the intensity of stimulation at all times.

## Results

### Acute Stimulation

**FIGURE 2:**
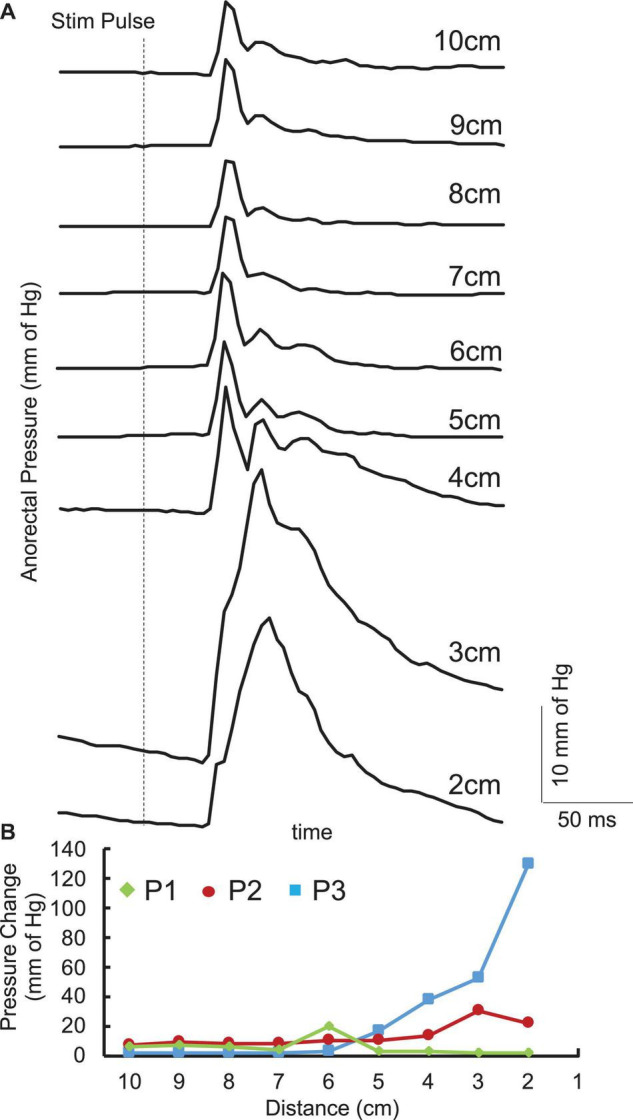
**(A)** Average (*n* = 5) pulses spinally evoked pressure change with singe pulses at 150 mA in the anorectal region 10 cm to 2 cm from the anus from a representative SCI (P1) patient. **(B)** Pressure changes across the anorectal region 10 cm to 2 cm from the anus from the three patients enrolled in the study.

Single pulses delivered over the lumbosacral spinal cord generated contractions in the anorectum of all three acute stimulation patients. The latency of contraction varied from 100 to 200 ms post stimulation pulse. The response amplitude varied based on the location. In addition, responses between 4 and 6 cm demonstrated a more complex response consisting of a second component with a longer latency (200–300 ms) (Figure 2A). However, location that generated the maximal amplitude occurred either between 1 and 3 cm or between 4 and 6 cm (Figure 2B).

**FIGURE 3:**
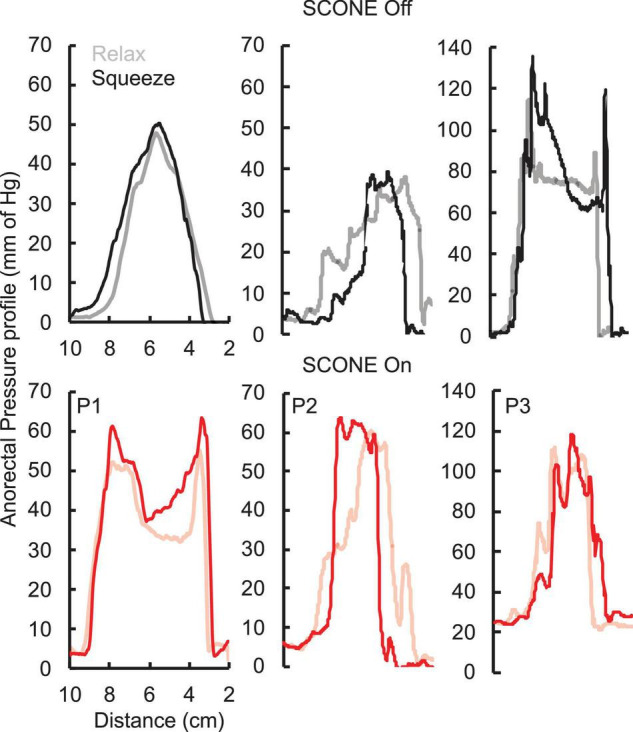
Anorectal pressure profile without (black) and with (ref) SCONE™ at T11 from the three SCI patients while the patients were relaxed (light) or squeezing (bold) their anus trying to assist a bowel movement.

The pressure profile across the anorectal region peaked between 4 and 6 cm (Figure 3) in all patients while the patients were relaxed and while they attempted to push as if to attempt a bowel movement. With tonic sub-motor threshold stimulation at T11 and L1, the pressure profile demonstrated a more consistent curve with high responses occurring between 3 and 8 cm. In addition, while the patient attempted to empty their bowels, the amplitude of response further increased between 3 and 8 cm. The change in pressure across the profile was higher while the patient was attempting to defecate and with the stimulation On (Figure 3).

**FIGURE 4:**
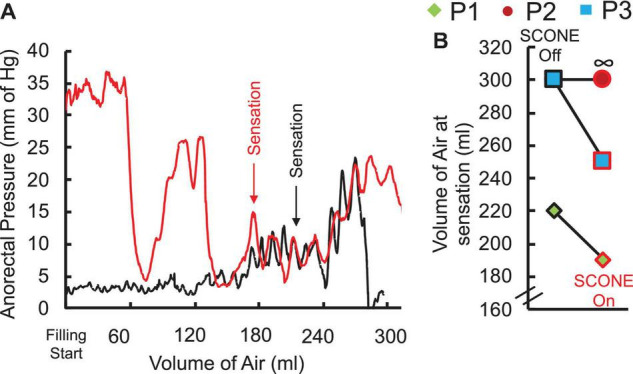
**(A)** Anorectal pressure recorded while manually filling the rectal balloon with air without (black) and with (red) spinal stimulation at T11 in a representative SCI patient (P1) AIS A at C5 without and with SCONE™. Note the sensation of filling appears at lower volume with compared to without SCONE™ (190 ml vs. 220 ml). **(B)** Threshold of sensation without and with SCONE™ from the three patients enrolled in the study. ∞ represents a case when the patient was unable to detect the filled air (mm Hg).

Without stimulation, one patient was able to sense a filled balloon in the rectum, whereas two patients were unable to sense it up to a volume of 300 ml, which was established as a safe upper limit by the clinician (Figure 4A). Upon initiation of tonic sub-motor threshold stimulation at T11 and L1, the sensate patient reported rectal sensation at a lower balloon volume, and one of the insensate patients reported new onset sensation with balloon distention of the rectum (Figure 4B).

### Chronic Stimulation

**FIGURE 5:**
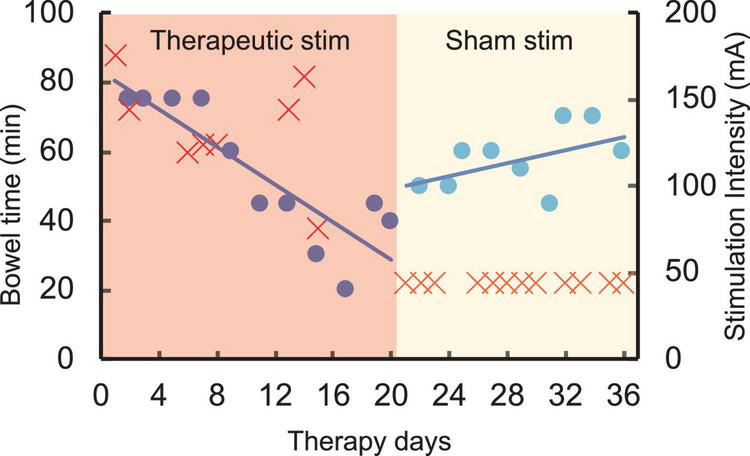
Time required to complete bowel program (blue) in an SCI patient (T4 AIS A) over 35 therapy days with varying intensities of stimulation (red). Note the decrease in bowel time over the first 18 days during higher intensity therapeutic stimulation (red region). However, the bowel program time increased close to baseline during the second half of the study with sham stimulation (yellow region).

The chronic stimulation patient was asked to record the amount of time needed to complete her bowel program. Prior to initiation of the study and during the first few days, her bowel program time was consistently approximately 75 mins. After completing 1 week of daily stimulation, her bowel program time reduced to 15 mins. Over the course of the next 18 days, sham stimulation was delivered, and her bowel program time increased to between 45 and 65 mins (Figure 5). No adverse effects of stimulation were reported by the patient or noted by the study staff.

## Discussion

In this pilot study we demonstrate that spinal neuromodulation can acutely change motor and sensory function of the anorectum. Additionally, in a case report of one patient, we show that spinal neuromodulation delivered over the course of several weeks may improve bowel function by decreasing the time required to perform a bowel program. SCONE™ is a non-invasive neuromodulation modality initially developed to promote lower extremity functional recovery after SCI. In our previous work, we have demonstrated that SCONE™ also appears to facilitate changes in lower urinary tract function in SCI individuals, promoting higher bladder capacity and decreasing detrusor overactivity (Gad P. N. et al., 2018; Kreydin et al., 2020). Although the bowel and the bladder differ markedly in function and control, their spinal and peripheral innervation share common origins. Parasympathetic innervation of both the anorectum and lower urinary tract (LUT) originates in the sacral parasympathetic nucleus located at the S2–S5 levels of the spinal cord (T12-L1 vertebral level). Onuf’s nucleus, which serves as the origin of the pudendal nerve, is located at the same level of the spinal cord and provides innervation of the skeletal external urethral and anal sphincters. Finally, sympathetic innervation of the anorectum and the LUT arises in the sympathetic chain at spinal levels T11-L2 (vertebral levels T7–T11) (de Groat et al., 2015; Callaghan et al., 2018). Thus, stimulation at levels T11 and L1 exerts an effect on all three components of the nervous system involved in bladder and bowel regulation. Just as with locomotion and LUT function, we hypothesize that spinal neuromodulation at these levels provides a signal to the relevant spinal centers, separated from the brain and brainstem by an injury, and allows acute and chronic changes to anorectal activity, that eventually lead to improvement in function.

An increase in anorectal pressure in response to SCONE™ was an effect we observed in each participant, with two participants exhibiting the strongest response in the region of the anal canal. This region is the location of the external (EAS) and internal (IAS) anal sphincters, with the puborectalis muscle located more proximally and giving rise to the former (Kaiser and Ortega, 2002). Thus, one of the two participants exhibited the strongest response at the level of the puborectalis (3.5 cm above the anal verge), while the other exhibited the strongest response at the level of the superficial EAS (0.5–2 cm above the anal verge), suggesting the pudendal nerve as the ultimate element that mediates the response as part of a spinal reflex arc (see discussion below). Other authors have demonstrated similar effects in response to transcutaneous magnetic stimulation of the sacral regions of patients with SCI. Morren et al. (2001) delivered transcutaneous magnetic stimulation laterally to the midline at S2–S4 vertebral levels, targeting the sacral spinal nerves as they exit the sacral foramina, and hypothesized that the strong pressure response noted in the anal canal was due to direct excitation of this nerve. An elevation in rectal pressure was also seen with transcutaneous magnetic stimulation of the cauda equina (L3–L5 vertebral levels) in studies by Lin et al. (2001) (both healthy controls and patients with SCI) and Shafik and El-Sibai (2000) (healthy individuals only). Interestingly, one participant exhibited the strongest pressure response in a more rostral location of the rectum, 6 cm above the anal verge. This location is considerably proximal to the skeletally innervated puborectalis and EAS, suggesting that this increase in pressure may be mediated by excitation of the autonomic nerves responsible for rectal function. Morren et al. (2001) also noted changes in rectal pressure (i.e., proximal to the puborectalis and the EAS) with transcutaneous magnetic stimulation and hypothesized that this was likely due to a reflex arc, whereby stimulation of large caliber somatic sensory nerves led to the excitation of autonomic fibers at the level of the lumbosacral spine.

While the exact neural structure, with which spinal neuromodulation interacts, remains to be determined, the relatively prolonged latencies and the multipeak nature of the pressure response suggest that the effect is not due to the direct stimulation of the pudendal nerve (causing anal sphincter contraction) but rather to a polysynaptic or reflex-mediated phenomenon. A similar response was noted by Fowler et al. (2000) and Schurch et al. (2003), when the S3 nerve root was percutaneously stimulated in able-bodied and SCI participants, respectively. A direct motor response would be expected to occur at a sub-10 ms timescale; on the other hand, the response we observed occurred at least 100 ms after pulse adminstration with a second component occurring up to 300 ms after. Similarly, a pudendo-anal reflex would be expected to occur with a latency of less than 40 ms (Cavalcanti Gde et al., 2007). Although latencies observed in this study could be mediated by an afferent spino-bulbospinal mechanism, this is less likely as the spinobulbar pathways are expected to be disrupted after an SCI. Nonetheless, it is possible that spinal neuromodulation promotes communication along damaged but anatomically persistent pathways. The other possibility is that the response we observed is mediated by a reflex of spinal origin. The latter possibility is supported by the persistence of the response even in patents with AIS A injuries both with acute and chronic stimulation.

In other applications of spinal neuromodulation, it was noted that stimulation improves not only motor activity, but also sensory function in patients with SCI (Gerasimenko et al., 2016; Gad P. N. et al., 2018), stroke and multiple sclerosis (Kreydin et al., 2020), and children with cerebral palsy (Edgerton et al., 2021; Gad et al., 2021a). Here we observed a similar phenomenon, whereby rectal sensation changed during application of stimulation, enabling two of the three participants to sense inflation of a rectal balloon at a lower volume than in the absence of stimulation. The mechanism by which spinal neuromodulation alters conscious sensation is not fully understood. As in our experience with urinary storage and locomotion, however, we hypothesized that SCONE™ may activate intact (but dormant) neural fibers at the injury site even in cases of functionally complete SCI and promote restoration of conscious sensation in a retrograde fashion. Alternatively, SCONE™ may lead to the excitation of sympathetic fibers, thus allowing rectal distention to elicit a sympathetic response at a lower volume. The participant may then appreciate this response as “rectal sensation.” Although it is unclear why Participant 2 did not exhibit this change in sensation, it is possible that this individual had a more complete injury and the reflex arc between rectal sensory fibers and the sympathetic chain/higher sensory centers was more significantly disrupted.

Finally, the finding that the participant who underwent daily SCONE™ sessions noted a gradual decrease in the time required to complete the bowel program suggests that SCONE™ stimulation may be a useful technique to promote functional improvement in patients with NBD. Several previous studies have assessed neuromodulation as a means of improving bowel function after SCI. Tsai et al. (2009) studied seven patients with supraconal SCI and assessed the effect of a 3-week course of daily transcutaneous magnetic stimulation at T9 and L3 on bowel function. A statistically significant decrease in colonic transit time and an improvement in questionnaire-measured bowel function were noted. Lin et al. (2001) reported similar findings in subjects who underwent transcutaneous magnetic stimulation of the cauda equina and exhibited a significant decrease in colonic transit time. In addition, subjects reported restoration of some sensory function and decreased reliance on digital stimulation to empty their bowels (Lin et al., 2001). Implantable sacral neuromodulation (Medtronic Interstim^®^, Minnesota, MN, United States) was assessed as a means to improve bowel function in patients with incomplete SCI by Jarrett et al. (2005) and was found to improve bowel continence and sensation of incomplete bowel emptying. Because patients with SCI suffer from abnormally prolonged bowel transit times and dyssenergic defecation, correction of these parameters would be expected to reduce the time required to complete a bowel movement. While the mechanism by which neuromodulation achieves these effects remains to be elucidated, we hypothesize that the stimulation signal interacts with neural fibers that remain after an injury to correct or improve end organ function.

Traditionally, non-invasive electrical spinal stimulation has been thought to be ineffective for stimulation of neural structures in the spinal cord because, unlike magnetic stimulation, significant signal attenuation occurs when an electrical impulse is applied to highly resistant tissues, such as skin, fat and bone that overlie the spinal cord. However, this study adds to the existing evidence that the unique dual frequency paradigm delivered by spinal neuromodulation can affect the function of neural structures in the spinal cord (Gerasimenko Y. et al., 2015). We hypothesize that the relatively high amplitude of stimulation afforded by spinal neuromodulation allows electrical charge to penetrate the investing tissues of the spinal cord and modulate the activity of spinal reflex arcs important for limb, lower urinary tract, respiratory and bowel control. Interestingly, interferential electrical current stimulation uses frequencies in the same order of magnitude as spinal neuromodulation and has been found to be effective in improving both clinical and physiological parameters of neurogenic bowel in pediatric patients with spina bifida (Kajbafzadeh et al., 2012).

While the results observed are significant, our study has from several limitations. First, the small number of patients limits the generalizability of the results. However, the activation of the anorectum across all three participants, and similar findings by other authors utilizing magnetic stimulation suggest that we observed a true physiologic effect. Secondly, our participants were fairly heterogeneous with varying levels and years post SCI. It is possible that with a more homogeneous cohort, more consistent effects of SCONE™ would have been observed. Future studies will need to determine how injury characteristics impact SCONE™ response and to identify biomarkers that modulate its effect. Finally, SCONE™ mediated improvement in bowel function after SCI requires further evaluation and confirmation in a rigorous sham-controlled setting.

## Data Availability Statement

The raw data supporting the conclusions of this article will be made available by the authors, without undue reservation.

## Ethics Statement

The studies involving human participants were reviewed and approved by the Rancho Research Institute. The patients/participants provided their written informed consent to participate in this study.

## Author Contributions

PG, EK, and VE designed the study. EK, PG, and HZ performed the experiments. All authors edited and approved the manuscript.

## Conflict of Interest

VE holds shareholder interest in Onward and hold certain inventorship rights on intellectual property licensed by The Regents of the University of California to Onward. VE, EK, and PG holds shareholder interest in SpineX Inc., and hold certain inventorship rights on intellectual property licensed by The Regents of the University of California to SpineX Inc. The remaining authors declare that the research was conducted in the absence of any commercial or financial relationships that could be construed as a potential conflict of interest.

## Publisher’s Note

All claims expressed in this article are solely those of the authors and do not necessarily represent those of their affiliated organizations, or those of the publisher, the editors and the reviewers. Any product that may be evaluated in this article, or claim that may be made by its manufacturer, is not guaranteed or endorsed by the publisher.
